# Large Animal Model for Development of Functional Restoration Paradigms Using Epidural and Intraspinal Stimulation

**DOI:** 10.1371/journal.pone.0081443

**Published:** 2013-12-05

**Authors:** Jan T. Hachmann, Ju Ho Jeong, Peter J. Grahn, Grant W. Mallory, Loribeth Q. Evertz, Allan J. Bieber, Darlene A. Lobel, Kevin E. Bennet, Kendall H. Lee, J. Luis Lujan

**Affiliations:** 1 Department of Neurologic Surgery, Mayo Clinic, Rochester, Minnesota, United States of America; 2 Mayo Graduate School, Mayo Clinic, Rochester, Minnesota, United States of America; 3 Department of Neurosurgery, Kosin University College of Medicine, Busan, Korea; 4 Department of Neurology, Mayo Clinic, Rochester, Minnesota, United States of America; 5 Department of Neurosurgery, Cleveland Clinic, Cleveland, Ohio, United States of America; 6 Division of Engineering, Mayo Clinic, Rochester, Minnesota, United States of America; 7 Department of Physiology and Biomedical Engineering, Mayo Clinic, Rochester, Minnesota, United States of America; Creighton University, United States of America

## Abstract

Restoration of movement following spinal cord injury (SCI) has been achieved using electrical stimulation of peripheral nerves and skeletal muscles. However, practical limitations such as the rapid onset of muscle fatigue hinder clinical application of these technologies. Recently, direct stimulation of alpha motor neurons has shown promise for evoking graded, controlled, and sustained muscle contractions in rodent and feline animal models while overcoming some of these limitations. However, small animal models are not optimal for the development of clinical spinal stimulation techniques for functional restoration of movement. Furthermore, variance in surgical procedure, targeting, and electrode implantation techniques can compromise therapeutic outcomes and impede comparison of results across studies. Herein, we present a protocol and large animal model that allow standardized development, testing, and optimization of novel clinical strategies for restoring motor function following spinal cord injury. We tested this protocol using both epidural and intraspinal stimulation in a porcine model of spinal cord injury, but the protocol is suitable for the development of other novel therapeutic strategies. This protocol will help characterize spinal circuits vital for selective activation of motor neuron pools. In turn, this will expedite the development and validation of high-precision therapeutic targeting strategies and stimulation technologies for optimal restoration of motor function in humans.

## Introduction

Limb movement is controlled by effector motor neurons and synaptic relays within the ventral horn grey matter of the spinal cord that receive inputs from higher brain centers. When signal transmission to these cells is interrupted, as in the case of spinal cord injury (SCI), permanent loss of sensorimotor and autonomic function can occur below the level of injury. Despite this, the cellular structure and neuronal networks remain intact and are capable of evoking and coordinating limb movements when electrically stimulated [1]–[4].

Recently, multiple studies of intraspinal stimulation have been successful in safely evoking coordinated limb movement and weight-bearing while improving fatigue-resistance in small animal models of SCI [5]–[9]. In previous studies, we demonstrated that wireless control of intraspinal microstimulation (ISMS) can be used to evoke graded and sustained contraction of hip, knee, and ankle muscles over prolonged periods of time in anesthetized SCI and spinally intact rats [10]. Additionally, fatigue resistant stepping has been demonstrated using intraspinal and epidural stimulation combined with systemic injection of excitatory neurotransmitter agonists [11]–[13]. Unfortunately, while existing small animal models are well established for performing proof-of-principle and preliminary studies, they are ultimately inadequate for determining clinical utility or for successful clinical translation of functional restoration techniques [14], [15]. Therefore, development of ISMS technology and functional stimulation paradigms for human application requires a model with appropriate dimensions and structure for accurate comparisons as well as evaluation of safety and efficacy. Functional hand movement has also been reported via cervical spinal stimulation in high-transection models of non-human primates [16]. However, primate models are costly and require long-term animal training. As such, there is a clear need for a large animal model that closely resembles the neuroanatomy of the human spinal cord and allows the development of novel, effective, safe, and cost-effective therapeutic paradigms. The neuroanatomy of the porcine spinal cord closely resembles that of humans [20] and represents a low-cost alternative to non-human primate models for the development and application of ISMS technology.

Herein, we present a standardized surgical model and spinal stimulation protocol in the adult white farm swine. This model allows the development and evaluation of novel therapeutic interventions and will ultimately help fill a critical gap in the translation of ISMS technology from small animal research to clinical human therapies. Ultimately, application of this model for the development and optimization of novel neural interface technology holds the potential to improve functional gains using spinal stimulation therapies and enhance quality of life for individuals with spinal cord injury.

## Methods

### Ethics Statement

All study procedures were approved by the Mayo Clinic Institutional Animal Care and Use Committee and were in accordance with the National Institutes of Health Guidelines for Animal Research (Guide for the Care and Use of Laboratory Animals).

### Animals

Six female domestic adult pigs (GeneticPork, Rochester, MN) aged 8–12 months and weighing between 25–35 kg were used for this model. Animals were kept in separate cages with visual access to each other in a controlled environment (constant temperature at 21°C and humidity at 45%) on a 12-hour light/dark cycle with *ad libitum* access to water. Animals were fed once daily.

### Animal Anesthesia

Animals were placed under general anesthesia with intramuscular telazol (5 mg/kg) and xylazine (2 mg/kg) for induction and 1.5–3% isoflurane for maintenance. Fentanyl (2–5 mg/kg/hr) was administered during surgery for analgesia. Heart rate (∼120 bpm), blood pressure (∼110/80 mmHg), and core body temperature (36–37°C) were continuously monitored to detect signs of pain or discomfort. Sufficient hydration was ensured by continuous intravenous (I.V.) saline infusion (75–150 ml/hr). Body temperature was maintained via heated blankets and warmed saline infusions. The short-acting non-depolarizing muscle relaxant vecuronium (0.057 mg/kg) was administered prior to spinal exposure to eliminate back musculature contractions during surgical manipulation.

### Lumbar Exposure

**Figure 1:**
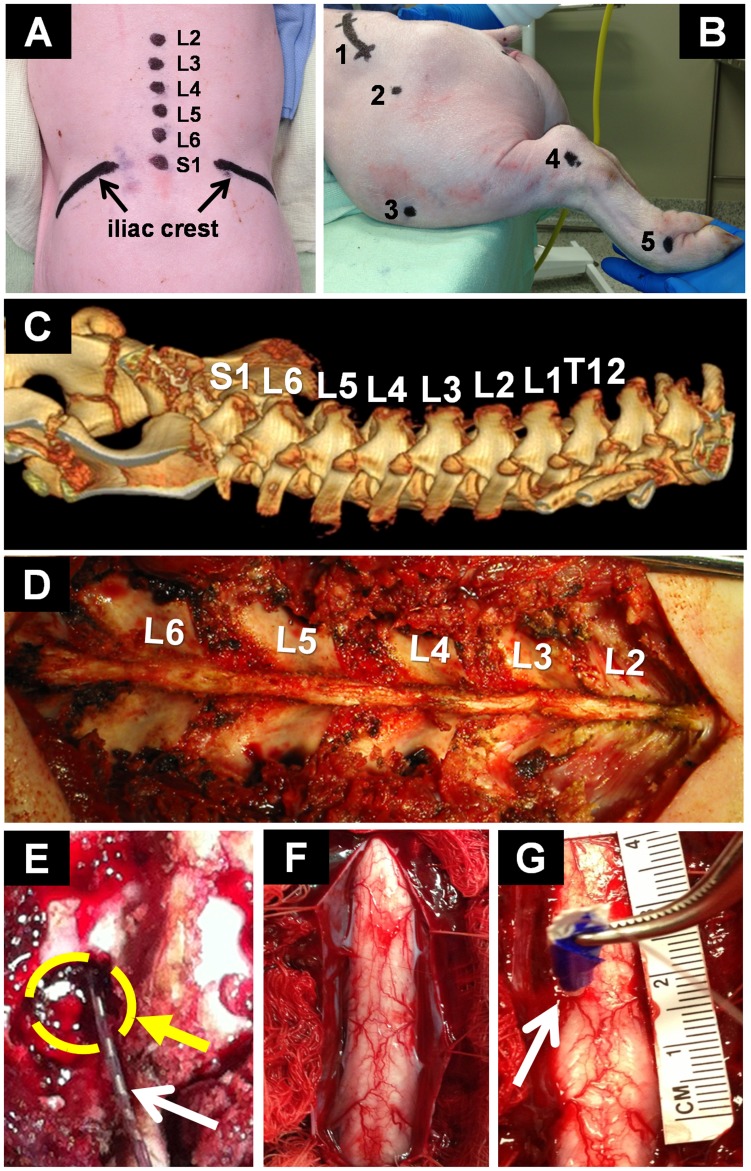
Experimental setup. (**A**) Anatomical landmarks (i.e., sacrum, iliac crest, and spinous processes) for localization of the lumbar spine (L2–S1); (**B**) Lateral view of anatomical motion analysis markers (1-Lateral iliac crest, 2-Trochanter major, 3-Patella, 4-Lateral malleolus, 5-Fourth metatarsal); (**C**) Pre-operative computed tomography: Lower thoracic vertebrae, lumbar vertebrae and sacrum; (**D**) Exposure of the lumbar spine; (**E**) Laminotomy at L5-left (circle) and epidural electrode (arrow); (**F**) Exposure of the spinal cord for intraspinal microstimulation; (**G**) Typical microelectrode implantation in the left hemicord (arrow). Insulating adhesive tape was used for protection of the electrode during forceps-insertion and color-coded to determine insertion depth (e.g., blue tape corresponded to an electrode depth of 6 mm). A reference ruler (centimeters) was used to determine cord dimensions and estimate electrode location.

Animals were positioned prone on an open-ended convex polymethyl-methacrylate cradle to allow stable surgical exposure, electrode insertion, and kinematic analysis of stimulation-evoked responses without requiring animal repositioning. Moderate kyphosis of the lumbar area facilitated the surgical procedure. The coat covering the surgical field was clipped using an electric shaver. Unlike the human lumbar spine, the porcine spine generally consists of six vertebrae. The lumbar enlargement resides between the third and fifth lumbar vertebrae (L3–L5) and was initially confirmed using pre-operative computed tomography (CT). Subsequent procedures were conducted using vertebral anatomical landmarks (i.e., the iliac crest and spinous processes) and intra-operative fluoroscopy (Fig. 1A,C). Location of the spinous processes allows orientation to midline. The spinous processes are connected via supraspinous and interspinous ligaments. A median skin incision from the second lumbar to the first sacral vertebral levels (L2–S1) was performed to allow access to the paraspinal musculature. Skin was retracted and the subcutaneous connective tissue was superficially incised to visualize the lumbodorsal fascia. The paraspinal musculature inserts at the facet joints and spinous processes via paravertebral tendons. The paravertebral tendons were bilaterally dissected and the paraspinous musculature was retracted and detached from the spinous processes and laminae using a combination of blunt preparation and monopolar electrocautery. Self-retaining retractors were inserted and progressively deepened with exposure to allow adequate visualization of the surgical field. Dissection in close proximity to the surface of the spinous processes reduced bleeding and facilitated subsequent bone removal. A small Cobb periosteal elevator was used to retract and sweep soft tissue from the spine, exposing the laminar surface (Fig. 1D). Bleeding was controlled using absorbable hemostats (Surgicel, Ethicon Inc, Somerville, NJ) and mono−/bipolar electrocautery.

### Epidural Stimulation

**Figure 2:**
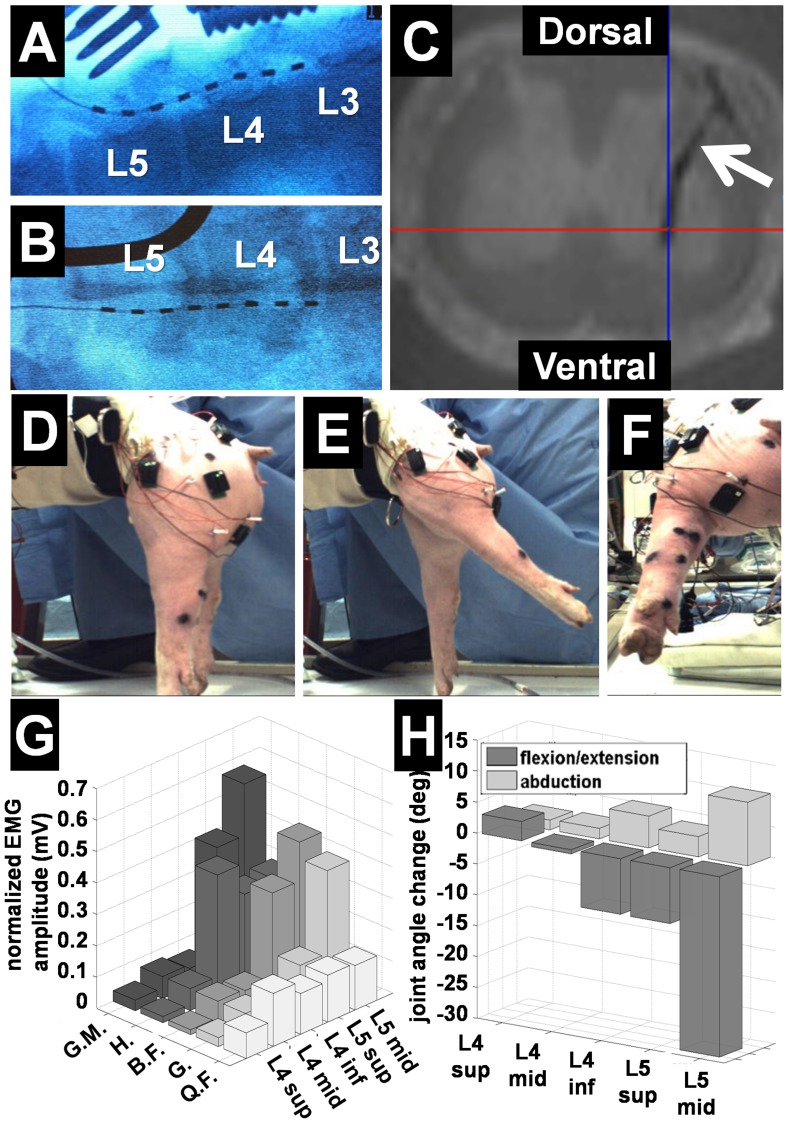
Targeting analysis and functional outcomes. (**A**) Epidural electrode placement confirmed by lateral intra-operative fluoroscopy; (**B**) Posterior-anterior intra-operative fluoroscopy; (**C**) *Ex-vivo* axial MRI of spinal cord showing ISMS electrode tract (arrow); (**D**) Experimental setup for kinematic analysis; (**E–F**) Typical hip extension evoked by ISMS at L5-segment; (**G**) Normalized intramuscular EMG amplitude as a function of spinal cord segment evoked by ISMS at 300 µA (n = 3); (**H**) Kinematic analysis of joint angle change evoked by ISMS as a function of spinal cord segment (n = 3). Abbreviations: G.M. = Gluteus medius, H = Hamstrings, B.F. = Biceps femoris, G = Gastrocnemius, Q.F. = quadriceps femoris.

A partial laminotomy was performed at the caudal end of the fifth lumbar vertebral level (L5) to allow insertion of epidural electrodes (Fig. 1E). The facet junction was identified and the bone thinned using a Horsley rongeur to expose the interior cortical margin. The spinal canal was then opened using a small angled curette by removing the remaining bone of the inferior L5 and superior L6 articular processes. The opening on the lower border of the lamina was extended using a 3 mm Kerrison rongeur. This decreased the risk of inadvertent injury to the dura mater during electrode insertion. The ligamentum flavum was incised to allow access to the dural sac. A cylindrical 8-contact epidural stimulating electrode (St. Jude Medical, St. Paul, MN) was inserted cranially through the laminotomy site (Fig. 1E). Accurate electrode contact placement was verified using intra-operative bi-planar fluoroscopy (Fig. 2A,B). We applied comprehensive bipolar stimulation at a frequency of 50 Hz with 200 µs pulse durations to all adjacent pairs of electrode contacts with amplitudes ranging from 100 to 1000 µA.

### Intraspinal Microstimulation (ISMS)

The spinal cord lumbar enlargement was exposed via complete laminectomies from L2 to S1, avoiding iatrogenic injury to large blood vessels, spinal cord tissue, nerve roots, and dura mater. First, the spinous processes were removed and the cortical margin exposed by thinning the laminar bone with a Leksell rongeur. Second, the bone shell was removed in a caudal to rostral direction from L2 to S1 using a 3 mm Kerrison rongeur while sparing the facet joints. Special attention should be placed not to extend the laminectomy beyond the lateral masses. Third, the ligamentum flavum and epidural fat pad were removed to expose the dural sac. Fourth, the dura mater was tented up with a sharp dura-hook or 5–0 silk suture and incised in the midline. The durotomy can be extended using a groove director and 15-blade or blunt micro-scissors while avoiding iatrogenic injury to the spinal cord. The dural flaps were bilaterally retracted using 5–0 silk sutures to expose the spinal cord parenchyma (Fig. 1F). Continuous saline irrigation was maintained to prevent cord dehydration.

Polyimide insulated (∼200 µm in diameter, A–M Systems, Sequim, WA) tungsten microwires were implanted into the porcine ventral spinal cord for stimulation (Fig. 1G). Insertion was guided by the dorsal fissure, bilateral posterior spinal arteries, and posterolateral dorsal root entry zones. Each microwire had a 250–400 µm exposed tip to create the stimulating electrode. Electrodes were labeled with tape using light microscopy prior to the procedure to mark targeted insertion depth and protect the electrode from damage during insertion. The tungsten microelectrodes allow a wide range of possible trajectories and insertion depths. Current-controlled bipolar spinal stimulation was applied through these electrodes using a four-channel portable stimulator (Mayo Investigational Neuromodulation Control System, MINCS) [17]. The stimulator was wirelessly-controlled in real-time using a Windows-based personal computer. We used a frequency of 50 Hz with 200 µs pulse duration and amplitudes of 50–300 µA. Microwire electrodes were orthogonally inserted at a trajectory that avoids the posterior spinal arteries to target lateral or medial ventral horn motor neurons, respectively. Insertion depth was varied from 2 to 6 mm from the posterior surface of the cord. A return tungsten microelectrode with a 10 mm exposed tip was implanted into the contralateral abdominal muscle. Electrode migration was prevented using a biocompatible cyanoacrylate adhesive applied to the dorsal surface of the spinal cord.

### Spinal Cord Injury Model

In addition to exposure of the lumbar spine, a complete spinal cord transection was performed at the fourth thoracic level (T4) following the surgical principles described previously. The target segment was localized using the most prominent spinous process (T2) and the turning point of the curvature from kyphosis to lordosis, usually at T7 or T8. This was verified using intra-operative fluoroscopy.

### Verification of Electrode Location

Placement of the epidural electrode was verified following epidural stimulation using intra-operative bi-planar fluoroscopy (Fig. 2A–B). In accordance with the recommendations of the American Veterinary Medical Association Guidelines on Euthanasia, animals were euthanized while still under anesthesia using a lethal dose of sodium-pentobarbital (50–75 mg/kg, I.V.) upon completion of the experimental protocol. Following euthanasia, the lumbar spinal cord (L2–L6) was surgically removed and immersed in a 4% paraformaldehyde solution. The resected spinal cord segment was imaged *ex vivo* using a 9.7 Tesla Magnetic Resonance Imager (Bruker 300 NMR) with a T2-weighted fast spin-echo (FSE) sequence. This allowed three-dimensional reconstruction and visualization of the intraspinal electrode tracts through the grey and white matter into the ventral horn (Fig. 2C).

### Analysis of Stimulation-evoked Motor Function

Kinematic and EMG analyses were performed during spinal stimulation. All animals were suspended in a custom-made system that allowed for electrode repositioning and unrestricted hindlimb movement across all degrees of freedom (Fig. 2D). Motion analysis markers were placed on the fourth metatarsal, ankle joint, knee joint, and iliac crest (Fig. 1B). Evoked joint movement was recorded using Basler ace GigE cameras (2048×1088 pixels, 100 fps) for parasagittal and posterior high-speed motion analysis (Contemplas, Templo, Kempten, Germany). Intramuscular EMG electrodes were implanted into the gluteus medius, quadriceps femoris, gastrocnemius, hamstrings, and biceps femoris muscles. Surface EMG sensors were placed over the gluteus medius, quadriceps femoris, hamstrings and biceps femoris muscles. Intramuscular and surface EMG data (common mode rejection ratio >80 dB and gain of 300) were recorded at 4000 Hz across a bandwidth of 20–450 Hz using a 16-bit Trigno system (Delsys, Boston, MA). The skin underlying the surface electrodes was shaved and cleaned to improve electrode–skin contact and reduce impedance. The surface EMG sensors were attached to the skin using a double-sided adhesive. For intramuscular EMG recordings, a reference electrode was inserted into the deep back musculature. EMG data were wirelessly recorded and transmitted to a Windows-based computer via Bluetooth technology for off-line analysis using Matlab (Mathworks, Natick, MA). Changes in joint angle were calculated by subtraction of joint position offsets before stimulation onset from the entire signal. To allow comparison across different muscles and subjects, EMG signal amplitude was determined by calculating the root mean squared (RMS) of the sampled signal and normalizing it to the maximum EMG amplitude recorded.

## Results

We tested our porcine model using epidural and intraspinal microstimulation techniques to evoke hind limb movement in six pigs. Preparation of the lumbar enlargement (including muscle dissection and bone removal from L2 to S1), radiographic identification of the stimulation targets, and electrode placement was achieved within 60 minutes of the initial incision. The time required for experimental testing depended upon the specific epidural or intraspinal stimulation protocol (i.e., stimulation paradigms, targets, and parameters) and can be safely extended under anesthesia. The maximum experimental time for the protocol described herein was 480 minutes. Injury to major blood vessels was the most common risk. However, bleeding was minimal (<0.3 L) and easily controlled using electrocautery, hemostats, gelfoam (Pfizer, New York, NY), and bone wax. Animal temperature was safely maintained within 36.5°C ±0.5°C throughout this time. One animal presented with borderline hypothermia (35°C) within the first 30 minutes of the procedure. Overall motion response to stimulation was decreased during this time. Normal temperature was restored using a combination of I.V. heated saline and heated blankets. There were no other major complications.

Optimal target location of the epidural electrode was readily achieved using lateral and posterior-anterior fluoroscopy (Fig. 2A,B). *Ex-vivo* imaging confirmed successful ISMS-targeting of the ventral horn gray matter (Fig. 2C). Epidural and intraspinal stimulation successfully evoked selective extension, flexion, adduction, and abduction of the knee and hip joints. Maximal hip extension was evoked via ISMS at the L5 level (Fig. 2H) with combined abduction/extension motion (Fig. 2E,F) and normalized EMG showed maximal activation of the gluteus medius, hamstrings, biceps femoris, and gastrocnemius muscles (Fig. 2G). Both epidural and intraspinal stimulation confirmed that spinal motor neurons controlling distal portions of the hind limb were located approximately 3 mm lateral from midline, while neurons controlling proximal portions of the hind limb were found approximately 1 mm from midline. Similarly, extensor neurons were found ventrally approximately 6 mm from the posterior surface of the cord and flexor neurons were localized more dorsally approximately 4 mm from the cord surface. Electromyography and kinematic analysis showed that joint angle change and EMG amplitude increased proportionally to stimulation amplitude. Furthermore, both types of stimulation were successful in evoking sustained tetanic muscle responses. The magnitude of these responses was not different for epidural and intraspinal stimulation (p>0.05). However, current amplitude thresholds required for evoking limb movement were five times lower for intraspinal compared to epidural stimulation. Initial muscle contractions in response to ISMS were generally observed at 50 µA compared to 250–300 µA for epidural stimulation. Motor response to epidural stimulation depended majorly on electrode location and distance between the contacts and spinal cord surface.

## Discussion and Future Directions

Traumatic injury to the spinal cord can result in permanent loss of motor, sensory, and autonomic function, significantly reducing quality of life for affected individuals. Functional electrical stimulation (FES) has been used clinically to restore lost function in paralyzed limbs by applying electrical currents either directly to the muscles or to peripheral nerves [3]–[5]. However, these techniques have had limited success due to early onset of muscle fatigue, limited degrees of freedom, large neuroprosthetic device size, and short battery life. Direct spinal stimulation techniques have been associated with recruitment of fatigue-resistant motor units in small-animal models [5], [7], [8], [18], [19]. Thus, the lower stimulation amplitudes associated with spinal stimulation, compared to conventional stimulation techniques, have the potential to increase battery life of implantable neuroprosthetic systems. However, these spinal stimulation techniques for restoration of motor function have only been attempted in small animal models, which are ultimately inadequate for the successful translation of functional restoration therapies into clinical application. As such, there is a significant need for a large animal model that not only allows evaluation of therapeutic efficacy and safety, but also optimization of targeting strategies and characterization of spinal circuits vital for selective activation of motor neuron pools.

The porcine spinal cord offers distinct advantages over other animal models for the development of functional restoration strategies following spinal cord injury. First, the spinal cord anatomy and physiology in the pig closely resemble the human spinal cord, thereby facilitating characterization of motor circuits. Several studies have shown that topographical organization of motor neurons and circuitry are maintained across species [20]–[22]. This allows the use of the porcine model to study therapeutic mechanisms and optimize the development of novel therapeutic paradigms for restoring function following spinal cord injury. Overall, the superior neuroanatomical resemblance of the porcine spine [23] and evolutionary conservation of topographical motor neuron networks, make this large animal model primed for expedited development of novel spinal therapies and evaluation of *in-vivo* long-term efficacy and safety. Second, its large spinal cord makes the porcine model ideal for optimizing targeting and delivery of stimulating electrodes. The model presented herein facilitates comprehensive three-dimensional topographical mapping of lumbar spinal cord neurons, thereby allowing for enhanced selectivity and control of motor function. Furthermore, this model has the potential for application in a variety of other research and clinical applications such as drug delivery systems for a host of spinal therapies including chemotherapy, gene therapy, and stem cell transplantation. It will also allow characterization of neuronal activation profiles, e.g. through electrophysiological recording or electrochemistry. Third, their low cost makes porcine models a feasible alternative to non-human primate models. However, having an appropriate animal model does not guarantee optimal translation of research developments into clinical therapeutic application. Differences in neuroanatomy, targeting strategy, and surgical skill can introduce significant variation in therapeutic outcomes. As such, it is paramount to standardize surgical and experimental protocols. This manuscript describes, step-by-step, the development of a large-animal model for the study of spinal cord physiology and restoration of function following neurologic injury and disease. Despite presenting significant advantages over existing models, the model described herein has inherent limitations. First, this work describes an acute model of spinal cord injury. Consequently, a stable model of chronic spinal cord injury will be required in order to establish the long-term feasibility of this type of therapeutic intervention. However, this model presents a good platform for mapping spinal neuron populations responsible for limb movement and for developing novel neuroprosthetic technology. Second, stimulation spillover can activate antagonist motor pools micrometers away from the electrode site [24]. Therefore, an automated stereotactic system needs to be developed for precise and accurate stimulation of target neurons. In turn, this will ensure subject safety while improving limb control and reliability of ISMS technology. Third, mapping locomotor regions of the spinal cord via rigid microelectrodes requires multiple penetrations. This extends surgery time and has the potential to damage spinal cord tissue [25]. For this reason, future studies should use multi-electrode arrays that may allow comprehensive and more precise activation of motor pools with potential for less tissue disruption. Fourth, the efficacy of microwire electrodes tends to diminish over time due to tissue reactions in vivo. Thus, in order to minimize tissue damage to the spinal cord, future work should be focused on developing flexible, implantable electrode arrays. Fifth, this model focuses on evoking simple motor responses. To this end, future work will need to focus on functional movements such as weight-bearing standing and ambulation that require independent but coordinated activation of muscles across multiple joints.

Successful application of spinal stimulation therapy in humans will require the use of versatile chronic neurostimulation approaches for optimal restoration of motor function. The standardized surgical procedure presented in this manuscript reduces the risk of inadvertent iatrogenic injury to spinal cord and nerve roots while allowing for relatively easy adaptation to other applications. Furthermore, this model has the potential to facilitate the development of novel clinical neural interfaces for chronic spinal cord stimulation, as well as the development and validation of techniques for long-term evaluation of efficacy and safety. In turn, the model and protocol presented herein represents an important step toward developing novel technology, which may ultimately lead to restoring independence and quality of life following spinal cord injury.
